# 5-(Adamantan-1-yl)-3-[(4-fluoro­anilino)meth­yl]-2,3-di­hydro-1,3,4-oxa­diazole-2-thione

**DOI:** 10.1107/S1600536813009823

**Published:** 2013-04-13

**Authors:** Abdul-Malek S. Al-Tamimi, Ahmed M. Alafeefy, Ali A. El-Emam, Seik Weng Ng, Edward R. T. Tiekink

**Affiliations:** a Department of Pharmaceutical Chemistry, College of Pharmacy, Salman bin Abdulaziz University, Alkharj 11942, Saudi Arabia; b Department of Pharmaceutical Chemistry, College of Pharmacy, King Saud University, Riyadh 11451, Saudi Arabia; c Department of Chemistry, University of Malaya, 50603 Kuala Lumpur, Malaysia; d Chemistry Department, Faculty of Science, King Abdulaziz University, PO Box 80203 Jeddah, Saudi Arabia

## Abstract

In the title compound, C_19_H_22_FN_3_OS, the dihedral angle between the inclined oxa­diazole and benzene rings is 52.7 (3)°. In the crystal, helical supra­molecular chains along [100] are sustained by N—H⋯S hydrogen bonds supported by methyl­ene–benzene C—H⋯π inter­actions. The crystal studied was an inversion twin with the fractional contribution of the minor component being 0.33 (14).

## Related literature

For biological background to adamantyl-1,3,4-oxa­diazole derivatives and for the structure of the phenyl derivative, see: Al-Tamimi *et al.* (2013 ▶).
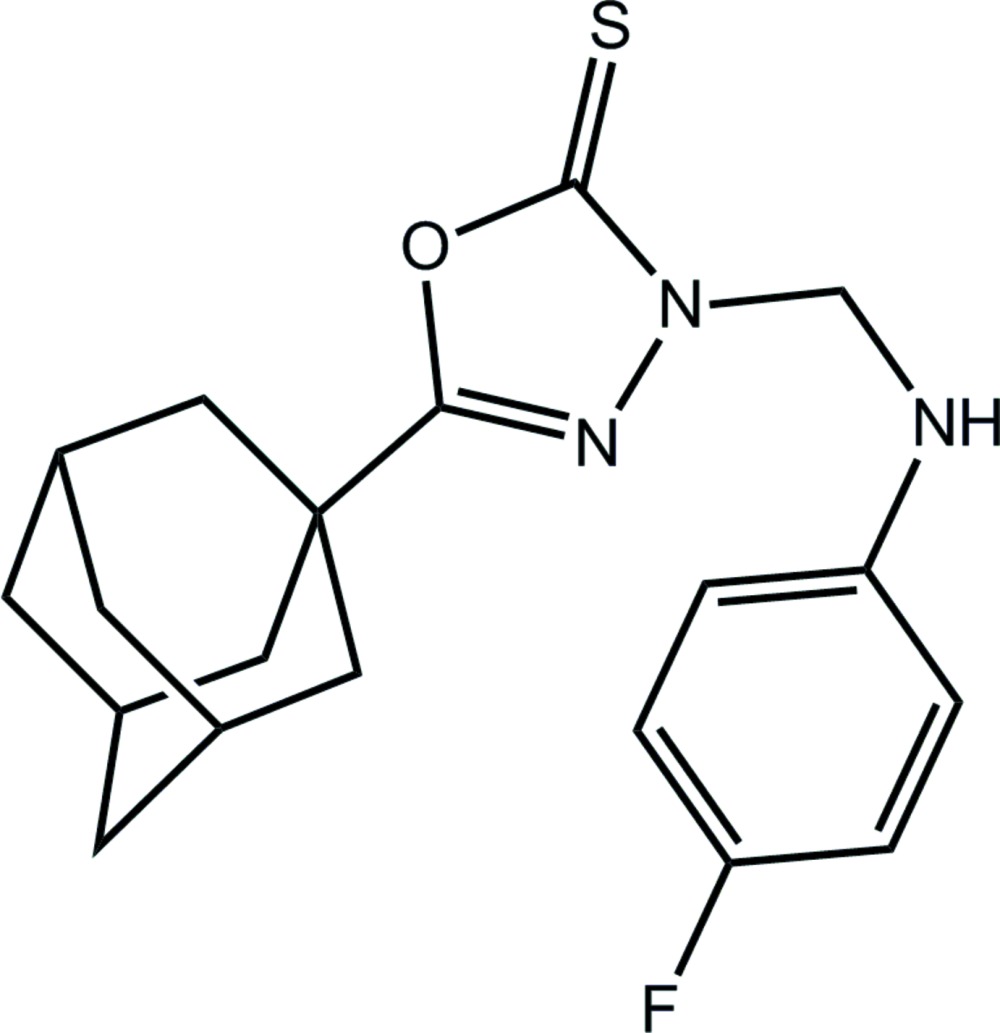


## Experimental

### 

#### Crystal data

C_19_H_22_FN_3_OS
*M* *_r_* = 359.47
Orthorhombic, 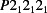

*a* = 7.1683 (8) Å
*b* = 10.6621 (11) Å
*c* = 23.592 (3) Å
*V* = 1803.1 (3) Å^3^
*Z* = 4
Mo *K*α radiation
μ = 0.20 mm^−1^
*T* = 295 K
0.30 × 0.10 × 0.10 mm

#### Data collection

Agilent SuperNova Dual diffractometer with an Atlas detector
Absorption correction: multi-scan (*CrysAlis PRO*; Agilent, 2011 ▶) *T*_min_ = 0.698, *T*_max_ = 1.000
7867 measured reflections
3902 independent reflections
2177 reflections with *I* > 2σ(*I*)
*R*_int_ = 0.043

#### Refinement

*R*[*F* ^2^ > 2σ(*F* ^2^)] = 0.063
*wR*(*F* ^2^) = 0.158
*S* = 0.98
3902 reflections
231 parameters
1 restraint
H atoms treated by a mixture of independent and constrained refinement
Δρ_max_ = 0.26 e Å^−3^
Δρ_min_ = −0.18 e Å^−3^
Absolute structure: Flack (1983 ▶), 1501 Friedel pairs
Flack parameter: 0.67 (14)

### 

Data collection: *CrysAlis PRO* (Agilent, 2011 ▶); cell refinement: *CrysAlis PRO*; data reduction: *CrysAlis PRO*; program(s) used to solve structure: *SHELXS97* (Sheldrick, 2008 ▶); program(s) used to refine structure: *SHELXL97* (Sheldrick, 2008 ▶); molecular graphics: *ORTEP-3 for Windows* (Farrugia, 2012 ▶) and *DIAMOND* (Brandenburg, 2006 ▶); software used to prepare material for publication: *publCIF* (Westrip, 2010 ▶).

## Supplementary Material

Crystal structure: contains datablock(s) global, I. DOI: 10.1107/S1600536813009823/hb7069sup1.cif

Structure factors: contains datablock(s) I. DOI: 10.1107/S1600536813009823/hb7069Isup2.hkl

Supplementary material file. DOI: 10.1107/S1600536813009823/hb7069Isup3.cml

Additional supplementary materials:  crystallographic information; 3D view; checkCIF report

## Figures and Tables

**Table 1:** Hydrogen-bond geometry (Å, °) *Cg*1 is the centroid of the C14–C19 ring.

*D*—H⋯*A*	*D*—H	H⋯*A*	*D*⋯*A*	*D*—H⋯*A*
N3—H3⋯S1^i^	0.87 (2)	2.61 (2)	3.475 (4)	172 (4)
C9—H9*A*⋯*Cg*1^ii^	0.97	2.90	3.800 (5)	154

Symmetry codes: (i) 
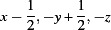
; (ii) 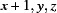.

**Fig. 1.**
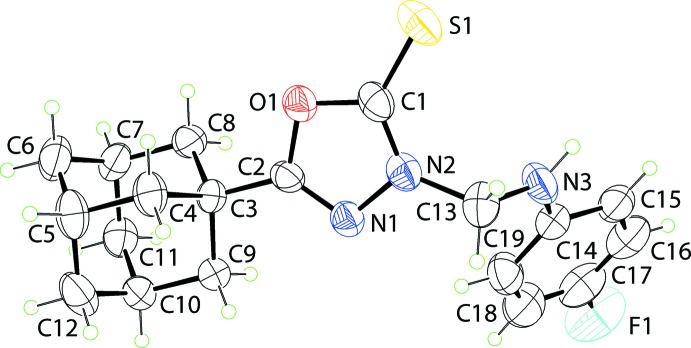
The molecular structure of (I) showing displacement ellipsoids at the 35% probability level.

**Fig. 2.**
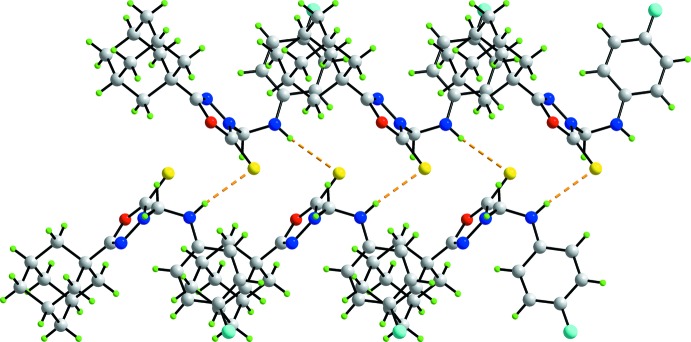
A view of the helical supramolecular chain along the a axis in (I), which is sustained by N—H···S hydrogen bonds shown as orange dashed lines.

**Fig. 3.**
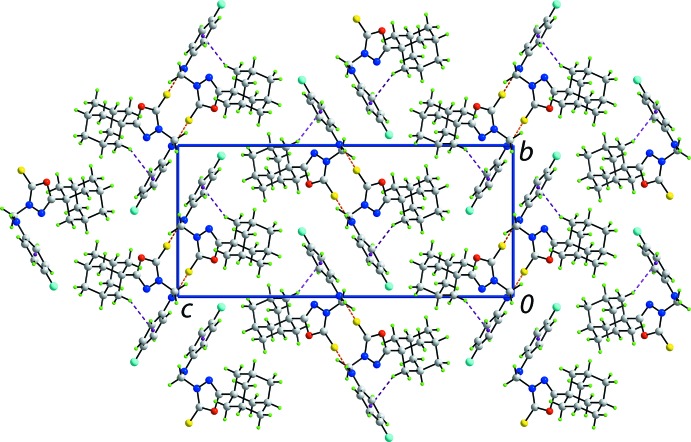
View of the unit-cell contents in projection down the a axis of (I). The N—H···S and C—H···π interactions are shown as orange and purple dashed lines, respectively.

## supplementary crystallographic information

### Comment

The biological background to adamantyl-1,3,4-oxadiazole derivatives and the crystal structure of the phenyl derivative is described in an accompanying paper (Al-Tamimi *et al.*, 2013). Herein, the crystal structure determination of the 4-fluoro derivative, (I), is described.

The 1,3,4-oxadiazole ring in (I), Fig. 1, is planar (r.m.s. deviation = 0.010 Å) and the thione-S1 atom lies 0.036 (1) Å out of this plane. The fluorobenzene ring lies to one side of this plane and forms a dihedral angle of 52.7 (3)°, resembling the situation in the parent compound (Al-Tamimi *et al.*, 2013). As for the parent compound, the thione-S1 and amine-N3—H atoms are *syn*, with the S1—C1···N3—H torsion angle being -5 (3)°.

In the crystal, helical supramolecular chains along [100] are formed through the agency of N—H···S hydrogen bonding, Fig. 2 and Table 1. Chains are consolidated methylene-C—H···π(benzene) interactions, Table 1, there being no specific interactions between the chains, Fig. 3.

### Experimental

A mixture of 5-(adamantane-1-yl)-1,3,4-oxadiazole-2-thiol (2.36 g, 0.01 mol), 4-fluoroaniline (1.11 g, 0.01 mol) and 37% formaldehyde solution (1.5 ml), in ethanol (15 ml), was stirred at room temperature for 2 h and allowed to stand overnight. The precipitated crude product was filtered, washed with water, dried, and crystallized from ethanol to yield 3.31 g (92%) of the title compound (I) as fine colourless crystals. *M*.pt: 453–455 K. Colourless prisms were obtained by slow evaporation of its solution in CHCl_3_-ethanol (1:1; 10 ml) held at room temperature.

### Refinement

The H-atoms were placed in calculated positions [C—H = 0.93 to 0.98 Å, *U*_iso_(H) = 1.2*U*_eq_(C)] and were included in the refinement in the riding model approximation. The N-bound H-atom was refined with the distance restraint N—H = 0.88±0.01 Å. The crystal is an inversion twin with the fractional contribution of the minor component being 0.33 (14).

### Crystal data

**Table:**

C_19_H_22_FN_3_OS	*F*(000) = 760
*M**_r_* = 359.47	*D*_x_ = 1.324 Mg m^−^^3^
Orthorhombic, *P*2_1_2_1_2_1_	Mo *K*α radiation, λ = 0.71073 Å
Hall symbol: P 2ac 2ab	Cell parameters from 1361 reflections
*a* = 7.1683 (8) Å	θ = 2.8–27.5°
*b* = 10.6621 (11) Å	µ = 0.20 mm^−^^1^
*c* = 23.592 (3) Å	*T* = 295 K
*V* = 1803.1 (3) Å^3^	Prism, colourless
*Z* = 4	0.30 × 0.10 × 0.10 mm

### Data collection

**Table:**

Agilent SuperNova Dual diffractometer with an Atlas detector	3902 independent reflections
Radiation source: SuperNova (Mo) X-ray Source	2177 reflections with *I* > 2σ(*I*)
Mirror monochromator	*R*_int_ = 0.043
Detector resolution: 10.4041 pixels mm^-1^	θ_max_ = 27.6°, θ_min_ = 3.0°
ω scan	*h* = −9→8
Absorption correction: multi-scan (*CrysAlis PRO*; Agilent, 2011)	*k* = −12→13
*T*_min_ = 0.698, *T*_max_ = 1.000	*l* = −30→30
7867 measured reflections	

### Refinement

**Table:**

Refinement on *F*^2^	Secondary atom site location: difference Fourier map
Least-squares matrix: full	Hydrogen site location: inferred from neighbouring sites
*R*[*F*^2^ > 2σ(*F*^2^)] = 0.063	H atoms treated by a mixture of independent and constrained refinement
*wR*(*F*^2^) = 0.158	*w* = 1/[σ^2^(*F*_o_^2^) + (0.0632*P*)^2^] where *P* = (*F*_o_^2^ + 2*F*_c_^2^)/3
*S* = 0.98	(Δ/σ)_max_ < 0.001
3902 reflections	Δρ_max_ = 0.26 e Å^−^^3^
231 parameters	Δρ_min_ = −0.18 e Å^−^^3^
1 restraint	Absolute structure: Flack (1983), 1501 Friedel pairs
Primary atom site location: structure-invariant direct methods	Flack parameter: 0.67 (14)

### Special details

**Table:**

Geometry. All s.u.'s (except the s.u. in the dihedral angle between two l.s. planes) are estimated using the full covariance matrix. The cell s.u.'s are taken into account individually in the estimation of s.u.'s in distances, angles and torsion angles; correlations between s.u.'s in cell parameters are only used when they are defined by crystal symmetry. An approximate (isotropic) treatment of cell s.u.'s is used for estimating s.u.'s involving l.s. planes.
Refinement. Refinement of *F*^2^ against ALL reflections. The weighted *R*-factor *wR* and goodness of fit *S* are based on *F*^2^, conventional *R*-factors *R* are based on *F*, with *F* set to zero for negative *F*^2^. The threshold expression of *F*^2^ > 2σ(*F*^2^) is used only for calculating *R*-factors(gt) *etc*. and is not relevant to the choice of reflections for refinement. *R*-factors based on *F*^2^ are statistically about twice as large as those based on *F*, and *R*- factors based on ALL data will be even larger.

### Fractional atomic coordinates and isotropic or equivalent isotropic displacement parameters (Å^2^)

**Table:**

	*x*	*y*	*z*	*U*_iso_*/*U*_eq_	
S1	0.06569 (15)	0.33471 (10)	0.03304 (5)	0.0836 (4)	
F1	−0.2851 (7)	−0.4312 (3)	0.12970 (14)	0.1582 (16)	
O1	0.3173 (4)	0.2563 (2)	0.10859 (11)	0.0620 (6)	
N1	0.3424 (4)	0.0519 (3)	0.09315 (12)	0.0587 (8)	
N2	0.2100 (4)	0.1064 (3)	0.05696 (13)	0.0611 (8)	
N3	−0.0577 (5)	−0.0053 (3)	0.01856 (17)	0.0778 (10)	
H3	−0.147 (5)	0.037 (4)	0.0020 (17)	0.098 (15)*	
C1	0.1962 (5)	0.2294 (3)	0.06489 (17)	0.0612 (9)	
C2	0.4025 (5)	0.1442 (3)	0.12294 (15)	0.0541 (8)	
C3	0.5497 (5)	0.1445 (3)	0.16727 (14)	0.0511 (8)	
C4	0.7151 (6)	0.2249 (4)	0.14706 (16)	0.0696 (11)	
H4A	0.7643	0.1911	0.1119	0.083*	
H4B	0.6736	0.3100	0.1400	0.083*	
C5	0.8671 (6)	0.2249 (4)	0.19230 (18)	0.0777 (13)	
H5	0.9729	0.2756	0.1793	0.093*	
C6	0.7903 (7)	0.2793 (4)	0.24685 (19)	0.0832 (13)	
H6A	0.7476	0.3644	0.2403	0.100*	
H6B	0.8879	0.2819	0.2753	0.100*	
C7	0.6290 (6)	0.1993 (3)	0.26791 (17)	0.0675 (11)	
H7	0.5808	0.2339	0.3035	0.081*	
C8	0.4746 (5)	0.1993 (3)	0.22315 (15)	0.0623 (10)	
H8A	0.4311	0.2843	0.2169	0.075*	
H8B	0.3701	0.1494	0.2363	0.075*	
C9	0.6160 (5)	0.0102 (3)	0.17771 (15)	0.0592 (9)	
H9A	0.5122	−0.0409	0.1905	0.071*	
H9B	0.6633	−0.0255	0.1427	0.071*	
C10	0.7691 (6)	0.0105 (4)	0.22242 (17)	0.0670 (11)	
H10	0.8114	−0.0757	0.2290	0.080*	
C11	0.6935 (7)	0.0645 (4)	0.27741 (17)	0.0738 (11)	
H11A	0.7900	0.0627	0.3062	0.089*	
H11B	0.5896	0.0140	0.2906	0.089*	
C12	0.9315 (6)	0.0890 (5)	0.2021 (2)	0.0875 (13)	
H12A	0.9801	0.0545	0.1670	0.105*	
H12B	1.0304	0.0875	0.2301	0.105*	
C13	0.1279 (6)	0.0329 (4)	0.01020 (16)	0.0736 (11)	
H13A	0.1337	0.0827	−0.0242	0.088*	
H13B	0.2040	−0.0412	0.0042	0.088*	
C14	−0.1079 (6)	−0.1144 (3)	0.04713 (15)	0.0643 (10)	
C15	−0.2947 (6)	−0.1525 (4)	0.04433 (18)	0.0758 (11)	
H15	−0.3804	−0.1057	0.0236	0.091*	
C16	−0.3509 (9)	−0.2588 (5)	0.0722 (2)	0.0968 (16)	
H16	−0.4748	−0.2843	0.0704	0.116*	
C17	−0.2266 (12)	−0.3262 (5)	0.1021 (2)	0.1003 (18)	
C18	−0.0461 (10)	−0.2929 (5)	0.1046 (2)	0.1024 (17)	
H18	0.0378	−0.3429	0.1245	0.123*	
C19	0.0173 (7)	−0.1844 (4)	0.07792 (17)	0.0835 (13)	
H19	0.1414	−0.1598	0.0809	0.100*	

### Atomic displacement parameters (Å^2^)

**Table:**

	*U*^11^	*U*^22^	*U*^33^	*U*^12^	*U*^13^	*U*^23^
S1	0.0517 (6)	0.0873 (7)	0.1118 (8)	0.0022 (6)	−0.0058 (6)	0.0342 (7)
F1	0.245 (5)	0.104 (2)	0.126 (2)	−0.040 (3)	0.038 (3)	0.021 (2)
O1	0.0485 (14)	0.0574 (14)	0.0800 (16)	−0.0034 (13)	−0.0044 (14)	0.0029 (13)
N1	0.0481 (18)	0.0599 (18)	0.0681 (18)	−0.0007 (16)	−0.0024 (15)	0.0012 (16)
N2	0.0510 (19)	0.0658 (19)	0.0666 (19)	−0.0018 (16)	−0.0026 (15)	0.0062 (16)
N3	0.050 (2)	0.075 (2)	0.108 (3)	−0.003 (2)	−0.021 (2)	0.013 (2)
C1	0.042 (2)	0.069 (2)	0.073 (2)	−0.006 (2)	0.0028 (19)	0.012 (2)
C2	0.0405 (19)	0.0489 (19)	0.073 (2)	0.0026 (17)	0.0036 (17)	0.0031 (18)
C3	0.0438 (19)	0.0481 (18)	0.0613 (19)	−0.0078 (17)	−0.0004 (16)	−0.0019 (16)
C4	0.052 (2)	0.079 (2)	0.077 (2)	−0.019 (2)	0.002 (2)	0.007 (2)
C5	0.052 (2)	0.098 (3)	0.084 (3)	−0.029 (2)	0.002 (2)	0.010 (2)
C6	0.083 (3)	0.070 (2)	0.097 (3)	−0.023 (3)	−0.026 (3)	0.001 (2)
C7	0.070 (3)	0.064 (2)	0.068 (2)	−0.015 (2)	0.001 (2)	−0.014 (2)
C8	0.053 (2)	0.057 (2)	0.077 (2)	−0.0014 (19)	0.0070 (19)	−0.006 (2)
C9	0.052 (2)	0.056 (2)	0.070 (2)	0.0021 (18)	−0.0048 (18)	−0.0083 (17)
C10	0.057 (3)	0.059 (2)	0.086 (3)	0.004 (2)	−0.012 (2)	−0.002 (2)
C11	0.073 (3)	0.074 (3)	0.075 (3)	−0.010 (2)	−0.007 (2)	0.004 (2)
C12	0.048 (2)	0.120 (4)	0.095 (3)	0.008 (3)	−0.001 (2)	−0.003 (3)
C13	0.076 (3)	0.079 (3)	0.067 (2)	−0.013 (2)	−0.011 (2)	0.005 (2)
C14	0.067 (3)	0.061 (2)	0.065 (2)	−0.002 (2)	−0.004 (2)	−0.009 (2)
C15	0.070 (3)	0.071 (2)	0.086 (3)	−0.006 (2)	0.001 (2)	−0.019 (2)
C16	0.105 (4)	0.086 (3)	0.099 (4)	−0.024 (3)	0.021 (3)	−0.024 (3)
C17	0.158 (6)	0.072 (3)	0.071 (3)	−0.020 (4)	0.014 (4)	−0.001 (3)
C18	0.138 (5)	0.086 (3)	0.083 (3)	0.003 (4)	−0.027 (3)	0.012 (3)
C19	0.086 (3)	0.084 (3)	0.080 (3)	0.000 (3)	−0.025 (2)	0.007 (3)

### Geometric parameters (Å, º)

**Table:**

S1—C1	1.643 (4)	C7—H7	0.9800
F1—C17	1.361 (5)	C8—H8A	0.9700
O1—C1	1.378 (5)	C8—H8B	0.9700
O1—C2	1.385 (4)	C9—C10	1.522 (5)
N1—C2	1.283 (4)	C9—H9A	0.9700
N1—N2	1.402 (4)	C9—H9B	0.9700
N2—C1	1.328 (5)	C10—C12	1.512 (6)
N2—C13	1.476 (5)	C10—C11	1.519 (5)
N3—C14	1.392 (5)	C10—H10	0.9800
N3—C13	1.406 (5)	C11—H11A	0.9700
N3—H3	0.874 (19)	C11—H11B	0.9700
C2—C3	1.486 (5)	C12—H12A	0.9700
C3—C9	1.529 (5)	C12—H12B	0.9700
C3—C4	1.538 (5)	C13—H13A	0.9700
C3—C8	1.539 (5)	C13—H13B	0.9700
C4—C5	1.526 (6)	C14—C19	1.374 (5)
C4—H4A	0.9700	C14—C15	1.401 (6)
C4—H4B	0.9700	C15—C16	1.371 (6)
C5—C6	1.515 (6)	C15—H15	0.9300
C5—C12	1.538 (6)	C16—C17	1.345 (8)
C5—H5	0.9800	C16—H16	0.9300
C6—C7	1.521 (6)	C17—C18	1.343 (8)
C6—H6A	0.9700	C18—C19	1.394 (6)
C6—H6B	0.9700	C18—H18	0.9300
C7—C11	1.526 (5)	C19—H19	0.9300
C7—C8	1.530 (5)		
			
C1—O1—C2	106.3 (3)	H8A—C8—H8B	108.2
C2—N1—N2	104.1 (3)	C10—C9—C3	109.5 (3)
C1—N2—N1	112.0 (3)	C10—C9—H9A	109.8
C1—N2—C13	126.8 (3)	C3—C9—H9A	109.8
N1—N2—C13	120.3 (3)	C10—C9—H9B	109.8
C14—N3—C13	123.7 (4)	C3—C9—H9B	109.8
C14—N3—H3	117 (3)	H9A—C9—H9B	108.2
C13—N3—H3	119 (3)	C12—C10—C9	109.7 (3)
N2—C1—O1	105.3 (3)	C12—C10—C11	109.6 (3)
N2—C1—S1	130.8 (3)	C9—C10—C11	109.6 (3)
O1—C1—S1	123.9 (3)	C12—C10—H10	109.3
N1—C2—O1	112.3 (3)	C9—C10—H10	109.3
N1—C2—C3	128.7 (3)	C11—C10—H10	109.3
O1—C2—C3	118.9 (3)	C10—C11—C7	109.8 (3)
C2—C3—C9	109.4 (3)	C10—C11—H11A	109.7
C2—C3—C4	109.3 (3)	C7—C11—H11A	109.7
C9—C3—C4	109.4 (3)	C10—C11—H11B	109.7
C2—C3—C8	110.8 (3)	C7—C11—H11B	109.7
C9—C3—C8	109.0 (3)	H11A—C11—H11B	108.2
C4—C3—C8	108.9 (3)	C10—C12—C5	109.8 (3)
C5—C4—C3	109.5 (3)	C10—C12—H12A	109.7
C5—C4—H4A	109.8	C5—C12—H12A	109.7
C3—C4—H4A	109.8	C10—C12—H12B	109.7
C5—C4—H4B	109.8	C5—C12—H12B	109.7
C3—C4—H4B	109.8	H12A—C12—H12B	108.2
H4A—C4—H4B	108.2	N3—C13—N2	115.2 (3)
C6—C5—C4	109.6 (4)	N3—C13—H13A	108.5
C6—C5—C12	110.0 (4)	N2—C13—H13A	108.5
C4—C5—C12	108.6 (4)	N3—C13—H13B	108.5
C6—C5—H5	109.6	N2—C13—H13B	108.5
C4—C5—H5	109.6	H13A—C13—H13B	107.5
C12—C5—H5	109.6	C19—C14—N3	122.8 (4)
C5—C6—C7	109.8 (3)	C19—C14—C15	119.5 (4)
C5—C6—H6A	109.7	N3—C14—C15	117.8 (4)
C7—C6—H6A	109.7	C16—C15—C14	119.8 (5)
C5—C6—H6B	109.7	C16—C15—H15	120.1
C7—C6—H6B	109.7	C14—C15—H15	120.1
H6A—C6—H6B	108.2	C17—C16—C15	119.9 (5)
C6—C7—C11	110.3 (4)	C17—C16—H16	120.0
C6—C7—C8	108.9 (3)	C15—C16—H16	120.0
C11—C7—C8	108.7 (3)	C18—C17—C16	121.3 (5)
C6—C7—H7	109.6	C18—C17—F1	119.6 (7)
C11—C7—H7	109.6	C16—C17—F1	119.1 (7)
C8—C7—H7	109.6	C17—C18—C19	120.9 (5)
C7—C8—C3	109.7 (3)	C17—C18—H18	119.6
C7—C8—H8A	109.7	C19—C18—H18	119.6
C3—C8—H8A	109.7	C14—C19—C18	118.5 (5)
C7—C8—H8B	109.7	C14—C19—H19	120.8
C3—C8—H8B	109.7	C18—C19—H19	120.8
			
C2—N1—N2—C1	1.2 (4)	C9—C3—C8—C7	59.9 (4)
C2—N1—N2—C13	171.3 (3)	C4—C3—C8—C7	−59.4 (4)
N1—N2—C1—O1	−2.0 (4)	C2—C3—C9—C10	179.1 (3)
C13—N2—C1—O1	−171.3 (3)	C4—C3—C9—C10	59.4 (4)
N1—N2—C1—S1	179.0 (3)	C8—C3—C9—C10	−59.6 (4)
C13—N2—C1—S1	9.8 (6)	C3—C9—C10—C12	−60.1 (4)
C2—O1—C1—N2	2.0 (4)	C3—C9—C10—C11	60.3 (4)
C2—O1—C1—S1	−179.0 (3)	C12—C10—C11—C7	59.6 (4)
N2—N1—C2—O1	0.1 (4)	C9—C10—C11—C7	−60.8 (4)
N2—N1—C2—C3	−177.7 (3)	C6—C7—C11—C10	−59.0 (4)
C1—O1—C2—N1	−1.3 (4)	C8—C7—C11—C10	60.4 (4)
C1—O1—C2—C3	176.7 (3)	C9—C10—C12—C5	60.8 (4)
N1—C2—C3—C9	−5.0 (5)	C11—C10—C12—C5	−59.5 (5)
O1—C2—C3—C9	177.4 (3)	C6—C5—C12—C10	59.2 (5)
N1—C2—C3—C4	114.8 (4)	C4—C5—C12—C10	−60.7 (5)
O1—C2—C3—C4	−62.9 (4)	C14—N3—C13—N2	−86.1 (5)
N1—C2—C3—C8	−125.2 (4)	C1—N2—C13—N3	−85.2 (5)
O1—C2—C3—C8	57.1 (4)	N1—N2—C13—N3	106.3 (4)
C2—C3—C4—C5	−179.7 (3)	C13—N3—C14—C19	11.1 (6)
C9—C3—C4—C5	−59.9 (4)	C13—N3—C14—C15	−169.5 (4)
C8—C3—C4—C5	59.1 (4)	C19—C14—C15—C16	−0.1 (6)
C3—C4—C5—C6	−60.2 (4)	N3—C14—C15—C16	−179.5 (4)
C3—C4—C5—C12	60.0 (4)	C14—C15—C16—C17	0.0 (6)
C4—C5—C6—C7	61.1 (5)	C15—C16—C17—C18	−1.2 (7)
C12—C5—C6—C7	−58.2 (5)	C15—C16—C17—F1	179.7 (4)
C5—C6—C7—C11	58.3 (5)	C16—C17—C18—C19	2.4 (8)
C5—C6—C7—C8	−60.9 (4)	F1—C17—C18—C19	−178.5 (4)
C6—C7—C8—C3	60.2 (4)	N3—C14—C19—C18	−179.4 (4)
C11—C7—C8—C3	−60.0 (4)	C15—C14—C19—C18	1.2 (6)
C2—C3—C8—C7	−179.7 (3)	C17—C18—C19—C14	−2.4 (7)

### Hydrogen-bond geometry (Å, º)

Cg1 is the centroid of the C14–C19 ring.

**Table:**

*D*—H···*A*	*D*—H	H···*A*	*D*···*A*	*D*—H···*A*
N3—H3···S1^i^	0.87 (2)	2.61 (2)	3.475 (4)	172 (4)
C9—H9*A*···*Cg*1^ii^	0.97	2.90	3.800 (5)	154

Symmetry codes: (i) *x*−1/2, −*y*+1/2, −*z*; (ii) *x*+1, *y*, *z*.
